# Serum leptin, adiposity, and their link to symptomatic hand osteoarthritis in individuals with radiological signs – A cross-sectional HALLOA cohort study

**DOI:** 10.1016/j.ocarto.2026.100879

**Published:** 2026-09-18

**Authors:** Malin Zimmerman, Noemi Raman, Margareta Törnblom, Maria LE. Andersson, Elisabeth Brogren

**Affiliations:** a Department of Orthopedics, Helsingborg Hospital, Helsingborg, Sweden; b Department of Translational Medicine – Hand Surgery, Lund University, Malmö, Sweden; c Department of Hand Surgery, Skåne University Hospital, Malmö, Sweden; d Lund University, Faculty of Medicine, Department of Clinical Sciences Lund, Rheumatology, Lund, Sweden; e Spenshult Research and Development Centre, Halmstad, Sweden; f Department of Environmental and Biosciences School of Business, Innovation and Sustainability, Halmstad University, Halmstad, Sweden

## Abstract

**Objective:**

To investigate the relationship between serum leptin levels and symptomatic hand osteoarthritis (OA) in individuals with radiographically confirmed hand OA.

**Method:**

This cross-sectional study included 90 individuals with radiographically confirmed hand OA. Symptomatic hand OA was diagnosed according to the American College of Rheumatology (ACR) criteria. Serum leptin levels, body mass index (BMI), and bioimpedance were measured. Participants reported pain intensity in the Numeric Rating Scale (NRS), self-reported upper-extremity disability (QuickDASH), number of bodily pain sites, and underwent pain pressure threshold (PPT) and grip strength measurements. Characteristics were compared between individuals with and without symptomatic hand OA. Binary logistic regressions were used to investigate the relationship between serum leptin levels and symptomatic hand OA. Adjustments were made stepwise for 1. age and sex, 2. adiposity, 3. radiographic severity of hand OA, and 4. PPT.

**Results:**

Symptomatic hand OA was diagnosed in 51 participants, whereas 39 did not have symptomatic hand OA. Participants with symptomatic hand OA had more hand pain, worse function, weaker grip, but also lower PPTs and more bodily pain. Metabolic factors did not differ between groups. Serum leptin levels were not associated with symptomatic hand OA. In a secondary finding, female sex was associated with symptomatic hand OA, an effect that was attenuated after adjustment for PPTs.

**Conclusion:**

Compared to those without symptoms, participants with symptomatic hand OA had lower PPTs and more widespread pain, consistent with features of central sensitization. While serum leptin was not independently associated with symptomatic hand OA, differences in pain sensitivity may contribute to the higher prevalence of symptomatic hand OA among women.

## 1 Introduction

The global prevalence of osteoarthritis (OA) is steadily increasing, partly due to an ageing population and rising risk factors such as obesity and a sedentary lifestyle [1]. The well-established association between OA and obesity, even in non-weight-bearing joints, suggests that systemic factors may contribute to disease mechanisms beyond mechanical load, particularly in hand OA [2].

Pain is the most prominent symptom of OA; yet it does not always align with clinical or radiological signs, as many individuals show joint changes without experiencing pain. Central pain sensitization has been proposed as a key mechanism underlying symptomatic OA and may partly explain this discrepancy [3].

Higher body mass index (BMI) and measures of central obesity have consistently been linked to more symptoms and higher pain scores in hand OA, independent of radiographic severity [[4], [5], [6]]. Longitudinal data indicate that higher BMI predicts worsening hand OA pain over time [7]. However, weight loss has not yet been shown to reduce the incidence of radiographic hand OA or resolution of hand OA pain, suggesting that the relationship may be more complex than a simple reversible effect of adiposity [8]. Adipokines such as leptin may modulate both peripheral inflammation and nociceptive processing, contributing to pain in OA beyond the mechanical effects of obesity [9]. This makes non-weight-bearing joints particularly suitable for study.

Serum leptin levels are closely related to BMI and have been studied in relation to hand OA with conflicting results [5,[10], [11], [12], [13]]. In the NEO cohort, serum leptin levels were associated with symptomatic hand OA and partially mediated the relationship between adiposity and hand OA prevalence [14]. However, a NHANES III cohort study found no association between leptin and symptomatic hand OA [10], and several studies report no association between leptin and radiographic hand OA [[11], [12], [13]]. However, in one study, leptin levels were associated with the intensity of hand OA pain [13] and another study suggested that the effect of BMI on hand OA pain was partly mediated by serum leptin levels [5]. Evidence also suggests sex-specific effects, with women showing higher leptin levels and stronger mediation of obesity-related OA risk and pain than men [[14], [15], [16]].

While structural joint damage reflects the morphological aspect of OA, symptom burden and pain sensitization represent its impact on daily life. Considering the findings above, it may be more relevant to investigate how serum leptin levels and obesity relate to symptom burden in hand OA rather than their association with structural joint damage visible on imaging [17].

Thus, this study aimed to investigate whether serum leptin levels were associated with the prevalence of symptomatic hand OA, adjusting for BMI and other relevant metabolic factors, among individuals with radiographic hand OA.

## 2 Methods

### 2.1 Participants

We used cross-sectional data from individuals with hand radiographs from the Halland Osteoarthritis (HALLOA) cohort (ClinicalTrials.gov NCT04928170) [18]. HALLOA is a longitudinal study of middle-aged individuals with knee pain at inclusion, designed to assess metabolic factors in OA development. Baseline examinations, samples, and measurements were conducted from 2016 to 2019.

In total, 306 individuals were included in the HALLOA cohort, of which 231 individuals (76 men and 155 women) underwent bilateral radiographic examination of the hands at their 2-year follow-up. Of these, 90 participants (25 men and 65 women) had radiographically confirmed hand OA as well as a complete clinical examination of the hands. They were included in the present cross-sectional cohort study.

### 2.2 Radiographic examination of hands

The radiographs were evaluated according to Kellgren-Lawrence (KL) by two independent examiners [19]. Joints were grouped into distal interphalangeal joints, proximal interphalangeal joints, and first carpometacarpal (CMC I) joints. A group was considered positive if at least one joint in the group was graded ≥2 according to KL. Radiographic hand OA was defined as having a KL score ≥2 in at least two of the three groups (DIP, PIP, and CMC I) in at least one hand [20]. The definition of radiographic hand OA severity was based on the number of involved joint groups, ranging from two to six groups (DIP, PIP, and CMC-I in both hands).

### 2.3 Clinical examination of hands

Bilateral grip strength was measured with the GRIPPIT instrument. A trained physiotherapist examined the hands according to the American College of Rheumatology criteria for symptomatic OA, as originally described by Altman et al. as “clinical hand OA” [21]. Individuals were classified as having symptomatic hand OA if, on examination, there was pain or stiffness in combination with soft tissue swelling in >2 of 10 joints, <3 swollen metacarpophalangeal (MCP) joints and soft tissue swelling in ≥2 distal interphalangeal joints or deformities in ≥2 of 10 joints [21].

### 2.4 Assessments of obesity and metabolic risk factors

Overweight/obesity was assessed by BMI (kg/m^2^). Overweight was defined as BMI ≥ 25 and obesity as BMI ≥ 30 [22]. Visceral fat area (VFA, cm^2^) and overall body fat (%) were measured using bioimpedance, Inbody 770 (Biospace South Korea). Waist circumference was measured at the umbilical level with a tape measure; three measurements were averaged. Central obesity was defined as ≥94 cm in men and ≥80 cm in women [23].

Blood pressure (BP) was measured after 5 min of rest. Hypertension was defined as self-reported previous diagnosis, assessed systolic BP ≥ 140 mmHg and/or diastolic BP ≥ 90 mmHg, or antihypertensive medication [24].

Fasting blood samples of triglycerides (mmol/L), total cholesterol (mmol/L), high-density (HDL) and low-density lipoprotein (mmol/L), glucose (mmol/L), and Hemoglobin A1c (HbA1c) (mmol/mol) were measured according to the current laboratory standards in Sweden. Fasting leptin levels were analyzed in serum using an ELISA method and performed as duplicates. Dyslipidemia was defined as self-reported previous diagnosis or medication for dyslipidemia, or triglycerides ≥ 1.7 mmol/L and/or HDL-cholesterol < 1.0 mmol/L (men) and <1.3 mmol/L (women) [25]. Diabetes was defined as self-reported previous diagnosis of diabetes mellitus type II, assessed fasting glucose ≥ 7.0 mmol/L or HbA1c ≥ 48 or taking oral antidiabetics and/or insulin [26]. The metabolic syndrome was defined according to the International Diabetes Federation's definition [27].

### 2.5 Questionnaires

Upper extremity function and symptoms were assessed using the short version of the Disabilities of the Arm, Shoulder and Hand (QuickDASH), a validated patient-reported outcome measure, scored 0–100, where a higher score represents worse problems [28]. The minimal important clinical difference for QuickDASH has been reported to be 18 points for patients with surgically treated CMC I OA [29].

Pain intensity was assessed using the Numeric Rating Scale (NRS), a validated outcome measure asking respondents to select a number 0 (no pain at all) to 10 (worst pain possible) that best represents their pain intensity during the last week [30]. Participants were asked to rate both overall bodily pain and their hand pain at rest, on motion without load, and on load. The minimal important clinical difference for NRS pain is 2–2.5 points for CMC I OA [31]. Participants also marked their painful areas on a pain manikin with 18 predefined pain regions [18].

Participants were asked to leave information on previous or current tobacco use and to fill in the Alcohol Use Disorders Identification Test. Weekly alcohol consumption was measured on a scale from 0 (no consumption) to 4 (frequent/daily drinking). Women were asked about hormone replacement treatment.

### 2.6 Pain Pressure Thresholds

PPTs were measured on eight predefined tender points out of the 18 points as a part of the definition of fibromyalgia [32] at the trapezius muscle (bilateral), second rib (right side), lateral epicondyle (right side), knees (bilateral at the fat pad), and gluteal area (bilateral) [18]. A hand-held algometer was used (1 cm^2^ probe; AlgoMed, Medoc, Israel). Force increased at ∼40 kPa/s from kPa from 0 to 1000 kPa or until the participant pressed the stop button. Two trials were performed at each tender point, at least 30 s apart. Participants were instructed: *“Press the button when you feel the first sensation shifting to pain”*. Five trained, blinded raters conducted the tests at inclusion and two at the two-year follow-up. Global PPT was defined as the mean across all eight sites.

### 2.7 Statistical analysis

Descriptive statistics were calculated for participants with and without symptomatic hand OA. Continuous variables are presented as means and standard deviations or median and interquartile range as appropriate, and categorical variables as proportions. Differences between groups were assessed using independent samples *t*-tests or Mann–Whitney *U* tests for continuous variables, and chi-square or Fisher's exact tests for categorical variables.

To examine associations between leptin levels and prevalence of symptomatic hand OA, we used binary logistic regression with symptomatic hand OA as the dependent variable. Covariates were chosen based on previous research and the clinical experience of the authors; no formal directed acyclic graph was constructed. Leptin was log10-transformed due to right-skewed distribution. Models were constructed in a stepwise approach.

• **Model 1** included log-leptin
• **Model 2** additionally adjusted for age and sex
• **Model 3** included log-leptin, age, sex, and body fat %
• **Model 4** included log-leptin, age, sex, body fat %, and radiological severity
• **Model 5** included log-leptin, age, sex, body fat %, radiological severity, and PPT

Model 3 (adjusted for age, sex, and body fat percentage) was designated as the primary analysis. In model 5, we included global PPT in an exploratory analysis to assess if central pain sensitization, reflected by lower PPTs, weakened the association between leptin and symptomatic hand OA. Only participants with complete data were included in the regression analyses.

To investigate the possible effect modification between leptin and adiposity, we tested an interaction term between log-leptin and BMI and stratified participants by BMI (<27 vs. ≥27). We chose a BMI of 27 as the cutoff point because it yielded reasonably balanced group sizes. A binary variable was created for this purpose. An interaction term was computed and entered into the regression model.

As a sensitivity analysis, each VFA, waist circumference, and BMI, was substituted for body fat % in models 3 and 4 to confirm that the estimate for log-leptin was not driven by the choice of adiposity measure.To test whether there was an interaction of sex, we created an interaction term. Parts of the statistical analysis strategy, including model specification and syntax writing, were developed with the assistance of an AI-based tool (ChatGPT, OpenAI). We performed all statistical analyses using IBM SPSS Statistics, version 30. This manuscript was edited for grammar and style using Grammarly (Grammarly Inc., San Francisco, CA, USA).

### 2.8 Ethics

All participants in the study provided their written consent, and the study was conducted in accordance with the Helsinki Declaration. The study has been approved by the Swedish Ethical Review Authority in Lund, Sweden (dnr 2016-229, 2017-253, 2018-602, 2020–04489, 2020–03866, and 2021-01837).

## 3 Results

From the original cohort, 251 participants had complete clinical examinations. Among the 90 participants with radiographic hand OA included in this study, 51 were classified as having symptomatic hand OA, and 39 were not. Fig. 1 illustrates the inclusion strategy employed in the present study. The groups were comparable with respect to age, sex, radiographic severity of hand OA, metabolic comorbidities, tobacco- and alcohol use, HRT medication, circulating leptin levels and obesity measured as BMI, body fat percentages, waist circumference and VFA (Table 1).

**Fig. 1:**
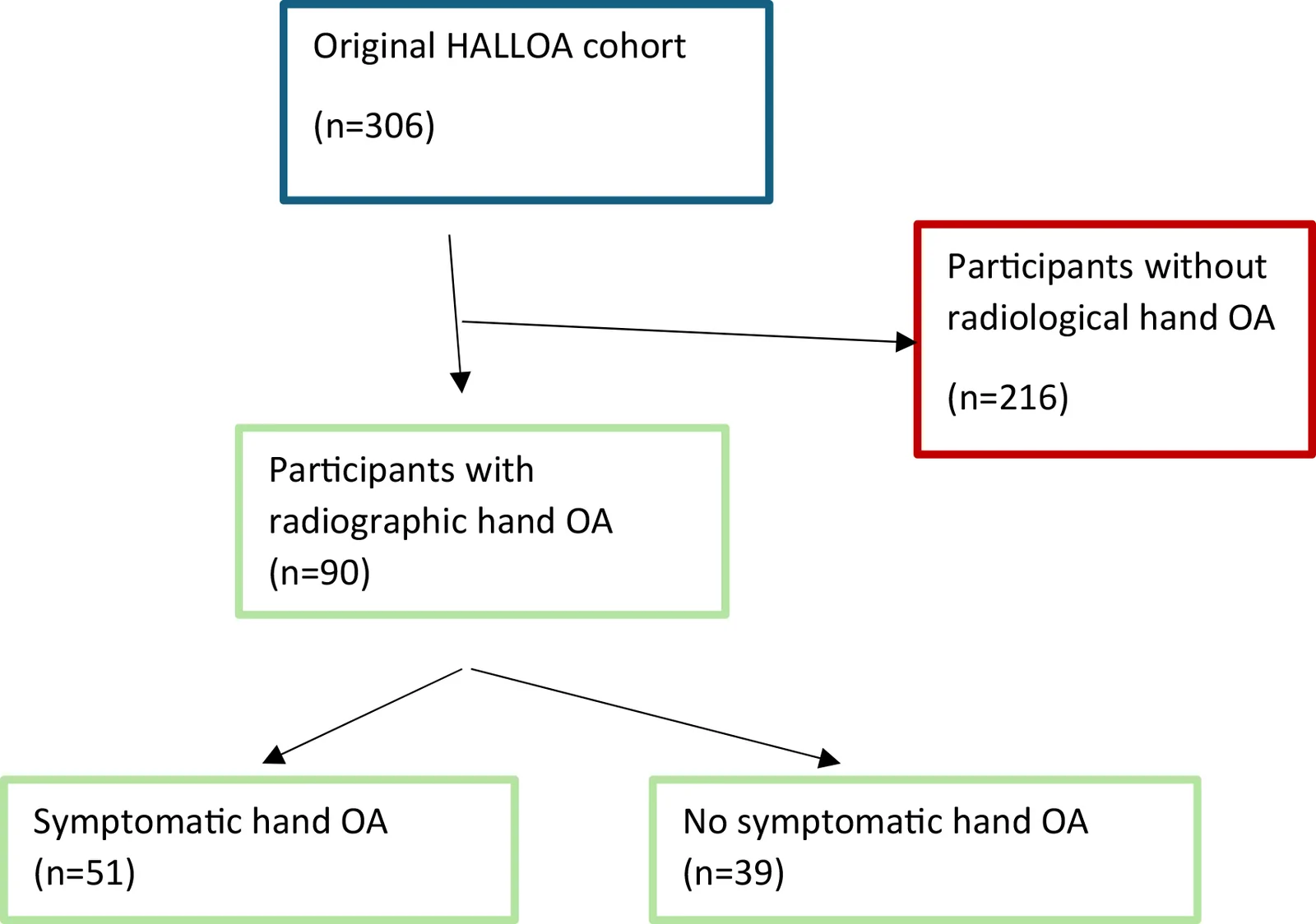
Flowchart describing the inclusion process for the present cross-sectional study.

**Table 1:** Characteristics of 90 participants with radiologic hand OA, divided into groups based on symptomatic hand OA or not.

	Symptomatic hand OA (n = 51)	No symptomatic hand OA (n = 39)	P-value
*Age, years*	58 ± 4	58 ± 5	0.59^a^
*Female sex, n (%)*	41 (80)	24 (62)	0.05
*Radiographic severity*	4 [2–5]	3 [2–4]	0.14^b^
*Average hand strength right hand, Newton*	232 ± 99	299 ± 106	**0.007**^a^
*QuickDASH, score*	18 [8–30]	8 [2–21]	**0.023**
*NRS hand pain at rest*	1 [0–2]	0 [0–1.75]	0.24^b^
*NRS hand pain on motion without load*	1 [0–4]	0 [0–2]	**0.033**^b^
*NRS hand pain on load*	3 [1–6.75]	0.5 [0–4.5]	**0.005**^b^
*NRS overall bodily pain*	3 [2–5]	1 [0–4]	**0.006**^b^
*No. of pain sites*	3 [1–6]	1 [0–4]	**0.011**^b^
*Global PPT, kPa*	328 ± 142	414 ± 158	**0.01**^a^
*BMI, kg/m*^*2*^	26.1 ± 3.7	27.4 ± 4.5	0.13^a^
*BMI, stratified, n (%)*			0.48^c^
*≤25*	27 (53)	17 (44)	
*26-30*	18 (35)	14 (36)	
*≥31*	6 (12)	8 (21)	
*Body fat, %*	32 ± 8	31 ± 9	0.68^a^
*Waist circumference, cm*	95 ± 11	99 ± 12	0.12^a^
Central obesity	42 (82)	37 (95)	0.17^c^
*VFA, cm*^*2*^	118 ± 48	123 ± 50	0.64^a^
*Leptin, ng/ml*	16.1 [6.0–30.2]	16.0 [6.1–27.3]	0.90^b^
*Dyslipidemia, n (%)*	8 (16)	8 (21)	0.55^c^
*Hypertension, n (%)*	30 (59)	30 (77)	**0.010**^c^
*Diabetes, n (%)*	1 (2)	0 (0)	0.40^d^
Metabolic syndrome, n (%)	14 (28)	14 (36)	0.21^c^
*Tobacco use, n (%)*			0.21^d^
*Never tobacco use*	35 (68.6)	21 (53.8)	
*Previous tobacco use*	10 (19.6)	14 (35.9)	
*Tobacco user*	5 (9.8)	3 (7.7)	
*Alcohol consumption*	1 [0–1]	1 [0–1]	0.88^b^
*HRT among females, n (%)*	3 (7)	0 (0)	0.18^d^

Data are presented as mean ± standard deviation if normally distributed or median [interquartile range] if non-normally distributed. Leptin data missing in four participants. Data on waist measurement and abdominal obesity missing in one participant. Radiographic severity presented as md [IQR] of number of involved joint groups raning from 2 to 6. HRT; Hormone replacement treatment.

a Independent *t*-test.

b Mann-Whitney *U* test.

c Chi square test.

d Fischer's exact test.

In the analyses of pain and functional outcomes, participants with symptomatic hand OA had weaker grip strength, greater NRS hand pain on motion and on load and worse self-perceived upper extremity function than those without symptomatic hand OA. In addition, participants with symptomatic hand OA had significantly lower global PPTs, together with higher overall bodily pain intensity and more pain sites (Table 1).

In the logistic regression analyses, log-transformed leptin was not significantly associated with symptomatic hand OA in any of the models (Table 2). In the sensitivity analysis, recalculating model 3 and 4 with each VFA, waist circumference, and BMI, the effect of leptin remained stable. Female sex was linked to symptomatic hand OA in all models, except the final one, where adding PPTs reduced its impact. Furthermore, the effect of sex was not significant in the interaction term (OR 1.11, 95% CI 0.10–12.38), indicating no evidence of a sex-specific effect of leptin on symptomatic hand OA in our dataset.

**Table 2:** Logistic regression models investigating the impact of serum leptin levels on hand OA, adjusting stepwise for age, sex, body fat and radiological severity in a cohort of 90 participants with radiological hand OA.

	Model 1 unadjusted	Model 2	Model 3	Model 4	Model 5
**LogLeptin**	0.88 (0.36–2.16)	0.46 (0.15–1.38)	0.39 (0.07–2.08)	0.40 (0.08–2.15)	0.53 (0.09–3.14)
**Age, years**		0.98 (0.89–1.08)	0.99 (0.91–1.10)	0.99 (0.89–1.11)	0.97 (0.88–1.08)
**Female sex**		**3.77 (1.20–11.86)**	**5.15 (1.50–17.64)**	**4.46 (1.27–15.61)**	2.45 (0.63–9.51)
**Body fat (%)**			1.00 (0.92–1.01)	1.00 (0.91–1.10)	0.97 (0.88–1.07)
**Radiological severity**				1.28 (0.90–1.84)	1.26 (0.87–1.82)
**PPT**					1.00 (0.99–1.00)

Radiological severity classified as number of joint groups involved (2–6). OA = osteoarthritis.

The interaction between leptin and BMI group was not statistically significant (OR 2.69, 95% CI 0.37–19.76).

## 4 Discussion

In this cross-sectional study of individuals with radiographic hand OA, participants with symptomatic hand OA had greater hand pain burden, worse upper-extremity disability, reduced grip strength, but also more widespread pain and lower PPTs compared to participants without symptomatic hand OA. These findings align with previous research suggesting that symptom severity in OA is not solely explained by structural joint changes but may involve altered pain processing mechanisms [3].

In the present study, serum leptin levels were not significantly associated with the presence of symptomatic hand OA. This is consistent with the NHANES III study, which found no link between leptin and symptomatic hand OA [10]. However, our results differ from the NEO cohort, where leptin mediated the relationship between adiposity and symptomatic hand OA prevalence [14]. The discrepancy may reflect differences in study design, population characteristics, or the definition of symptomatic or clinical OA. Consistent with our null finding, a recent study using machine learning in 143 patients with CMC I OA found no independent association between baseline plasma leptin and one-year patient-reported outcomes [33].

Given the small number of events relative to the number of covariates in the more complex models, the present study is likely underpowered to detect modest independent effects of leptin after adjustment, and the results should be interpreted as exploratory and hypothesis-generating.

Although the interaction between leptin and BMI was not statistically significant, the observed pattern indicates a higher probability of symptomatic OA with increasing leptin levels in participants with BMI ≥27, suggesting a possible BMI-dependent association. Also, there was a pattern towards higher leptin correlating with less likelihood of symptomatic OA in normal-weight individuals. This pattern is in line with previous reports indicating that metabolic factors may amplify pain in individuals with obesity [5,9]. The lack of statistical significance could be due to a limited sample size and warrants further investigation in larger cohorts.

Sex was associated with symptomatic hand OA, with women having higher odds than men, consistent with prior evidence of sex-specific differences in OA pathophysiology and potentially reflecting differences in adipokine profiles [[14], [15], [16]]. These findings underscore the importance of considering sex as a biological variable in OA research. The effect of hormone replacement treatment on clinical and radiological OA remains to be investigated, and the numbers in this cohort were too small to allow for any detailed investigations.

Our study also highlights the role of central pain sensitization in hand OA. Participants with symptomatic hand OA exhibited lower global PPTs and reported more widespread bodily pain, supporting the hypothesis that central pain mechanisms contribute to symptom burden beyond local joint pathology [3]. This may partly explain why weight loss interventions have not consistently improved hand OA pain despite reducing mechanical load [8]. Although central sensitization is most strongly associated with widespread bodily pain, evidence increasingly suggests that adipokines may relate to such pain phenotypes across inflammatory and degenerative joint disease; for example, higher leptin levels have recently been shown to be associated with chronic widespread pain in patients with rheumatoid arthritis, independent of BMI, age, and sex [34]. In our cohort, the addition of global PPT in the fully adjusted model did not substantially change the estimate for log-leptin, supporting the conclusion that there is no association between serum leptin and symptomatic hand OA in this sample. However, the association between female sex and symptomatic hand OA was substantially weakened, suggesting that the sex effect in this cohort is mediated by lower PPTs – i.e., through central sensitization and/or pain processing – rather than by an independent sex-specific pathway. This is consistent with previous evidence of sex differences in PPTs in healthy populations [35].

However, the present cohort may be subject to selection bias, as participants were recruited based on knee pain, which frequently coexists with hand OA. It is possible that the study sample represents a phenotype with a high degree of bodily pain, and the results may not be generalizable to a population with isolated hand OA. Although pain at rest was low in the symptomatic hand OA group (mean NRS 1), patients reported substantially higher pain during load compared with individuals without symptomatic hand OA. This suggests that the symptomatic group had clinically relevant symptoms despite low levels of resting pain. Nevertheless, the relatively mild symptom burden observed in our cohort compared with some previous studies may have reduced our ability to detect associations between leptin levels and symptomatic hand OA.

We acknowledge that the American College of Rheumatology criteria combine symptoms (pain, stiffness) with clinical examination findings (soft-tissue swelling, joint deformity), which share some conceptual overlap with the underlying radiological disease; the continuous pain and function measures reported alongside the dichotomous classification are intended to provide complementary perspectives on the symptomatic phenotype.

### 4.1 Strengths and limitations

Strengths include detailed phenotyping, standardized radiographic and clinical assessments, and measurement of multiple adiposity indicators. Limitations include the cross-sectional design, which precludes causal inference, and a relatively small sample size, limiting the power to detect interaction effects. There is a risk of a Type II error, as the study may have been underpowered to detect a modest independent effect of leptin on OA symptoms. We also did not have a healthy control group (no radiographic hand OA). Additionally, leptin was measured at a single time point, and other adipokines were not assessed. At inclusion, five trained, blinded examiners performed the PPT measurements, all following the same standardized protocol and training. Formal inter- and intra-rater reliability analyses were not performed in HALLOA.

The study sample was too small to allow for sex-stratified analyses, which could be an interesting direction for future research.

## 5 Conclusion

Compared to those without symptoms, participants with symptomatic hand OA had lower PPTs and more widespread pain, consistent with features of central sensitization. While serum leptin was not independently associated with symptomatic hand OA in this sample, differences in pain sensitivity may contribute to the higher prevalence of symptomatic hand OA among women.

## Author contributions

MA initialized and organized the study cohort, did the laboratory analyses and reviewed the manuscript. MZ, EB and MA conceptualized the study. MZ and EB analyzed all radiographs, analyzed the data, obtained funding and wrote the manuscript. NR wrote the first draft, participated in data analysis and reviewed the manuscript. MT perfomed clinical assessments of the participants, and reviewed the manuscript.

## AI use disclosure

Parts of the statistical analysis strategy, including model specification and syntax writing, were developed with the assistance of an AI-based tool (ChatGPT, OpenAI). This manuscript was edited for grammar and style using Grammarly (Grammarly Inc., San Francisco, CA, USA). The authors take full responsibility for the contents of this manuscript.

## Role of the funding source

This work was supported by grants from the regional agreement on medical training and clinical research (ALF) between Region Skåne and Lund University (MZ and EB) and Eva och Ingemars Nilssons minnesfond (MZ).

## Conflict of interest

The authors have no conflicts of interest to declare.
